# Thirty-first Annual Meeting of the United States Veterinary Medical Association

**Published:** 1894-11

**Authors:** 


### SOCIETY PROCEEDINGS.

### THIRTY-FIRST ANNUAL MEETING, UNITED STATES VETERINARY MEDICAL ASSOCIATION.

PHILADELPHIA PA., SEPTEMBER 18, 19, 20, 1894.

The thirty-first Annual Convention of the United States Veterinary Medical Association was held at the Academy of Natural Sciences, 19th and Race Sts., Philadelphia, Pa., on the 18th, 19th, and 20th of September. The Convention was called to order at 10.30 A.M., on the morning of the 18th, by President W. Horace Hoskins.

The Secretary then called the roll, and the following answered to their names: Drs. Allen, Barron, Bretherton, Bridge, Budd, Tait Butler, Cary, Clement, Curtice, Dixon, William Dougherty, Faust, Gill, Goenter, HagenBurger, Harger, Hoskins, Howard, Kooker, Lintz, Lusson, Lyman, H. J. McClellan, Martenet, E. Mayhew Michener, Middleton, W. B. E. Miller, Osgood, Paige, Parker, Pearson, J. B. Rayner, T. B. Rayner, Rhoads, J. L. Robertson, J. D. Robinson, Salmon, Schwarzkopf, Sellers, Trumbower, T. J. Turner, Williams, Winchester and Zuill.

Dr. Isaiah Michener as an honorary member.

Other members of the profession present: Drs. Jno. Adams, Philadelphia, Pa., Jno. R. Hart, Philadelphia, Pa., D. B. Fitzpatrick, Philadelphia, Pa., J. T. McAnulty, Philadelphia, Pa., George B. Rayner, Philadelphia, Pa., W. H. Ridge, Trevose, Pa., J. H. Wattles, Kansas City, Mo., H. J. Detmers, Columbus, Ohio, M. E. Conard, West Grove, Pa., C. B. Robinson, Washington, D. C.

The President then introduced Mayor Edwin S. Stuart of Philadelphia, who welcomed the delegates and members to Philadelphia, and referred to the appropriateness of the Convention convening in this city, which claimed to have established the first regularly organized Veterinary College, and referred to the growth of the veterinary profession in numbers, and recognized their great value to the community. He paid a high compliment to the Veterinary Department of the University of Pennsylvania, and with pride pointed to the existence in Philadelphia of one of the best and most complete municipal veterinary hospitals of any large city in this country.

The President then read his annual address, reviewing the work of the past year and the results obtained by the Association in their effort to more thoroughly discharge the responsibilities incumbent upon them as an organization.

The report of the Comitia Minora was then taken up and considered, and the following new members were elected to membership:

To honorary membership, Prof. Wesley Mills, Montreal, Canada.

To regular membership, Drs. C. H. Doepel, D.V.S., A.V.C., Mamaroneck, N. Y. Vouchers, E. B. Ackerman, W. H. Coates; David P. Fox, D.V.S., Chicago V. C., Sacramento, Cal. Voucher, R. A. Archibald; Thomas Maclay,

M.R.C.V.S., Glasgow, Scotland, Petaluma, California. Voucher, R. A. Archibald; H. F. Spencer, D.V.S., Chicago V. C., San Jose, California. Voucher, R. A. Archibald; H. J. S. Weicksel, V.M.D., University of Pennsylvania, V. D., Line Mountain, Northumberland Co., Pa. Voucher, S. J. J. Harger and Leonard Pearson; Walter C. Clevenger, V.S., Ont. V. C., Union City, Indiana. Voucher, J. O. Greeson; Frank H. Mackie, V.M.D., University of Pennsylvania, V.D., Fair Hill, Md. Voucher, A. W. Clement.

The action of the President and the other officers was approved in the settlement of the stenographer's bill at Chicago ; also the employment of one for the meeting of 1894.

The question referred to the Comitia Minora, asking what disposition would be made of an applicant for membership who would take an elective three years' course in a two years' school? The Comitia Minora decided that such an applicant would not be eligible to membership in the Association.

The President then announced the deaths of Drs. Charles B. Michener and John S. Saunders, upon which a motion was made to appoint a committee to draft suitable resolutions in regard to their death.

Committees appointed: Death of Dr. Michener—Drs. A. W. Clement, chairman, Jno. Faust, W. L. Zuill.

Death of Dr. Saunders—Drs. F. H. Osgood, Chairman, L. H. Howard and James L. Robertson.

The President then announced, on behalf of the Local Committe of arrangements, that there would be a lunch provided daily at 1 P. M. for all the members, delegates and visitors attending the Convention, and be served in one of the halls adjoining the meeting room ; also that arrangements had been made to carry all present by coaches to the Veterinary Department of the University of Pennsylvania, The Philadelphia Veterinary Sanitarium, and to the Zoological Garden at three-thirty on Thursday afternoon. The Zoological Society of Philadelphia, having extended a courteous invitation to the Convention to visit the gardens in a body.

The Secretary then read a number of letters acknowledging the high appreciation of those whom the Association at their Congress had conferred upon them honorary membership in the Congress, and the honorary membership of the Association.

After accepting the proposition of one of the leading photographers of the city to take a group photograph of the members without any cost to them, the meeting proceeded to consider the various reports of the standing committees.

The question of changing the initiation fee and dues was then taken up and in conjunction therewith the Secretary asked, on behalf of the Comitia Minora, for an expression of opinion of the Association as to what should be done with those members who declined to pay the assessment. After some discussion, it was decided that they should be classed among those in arrears for dues, and would be subject to the same penalty on failing to pay the same. The question was again raised as to the Association's right to levy the same, which was fully covered by the by-laws. This brought up the question as to the need of increasing the initiation fee and dues. and after considerable discussion it was decided that the initiation fee would remain at $5.00, but the annual dues after '94 would be $5.00 per year. For the information of the members the President stated that we were practically handing back to every member his dues in the annual copy of our transactions, they costing $2.00, which left no money for the other expenses of printing, etc., after which the Convention adjourned until 2 P. M.

On reconvening at 2 P. M., the report of the Committe on Intelligence and Education was called for, and was read by Dr. Cary, the Chairman. The various points brought up by the different members of the committee were then discussed by Drs. Williams, Schwarzkopf, Paige, Tait Butler, Salmon, Faust, Clement, Dixon, Pearson, Osgood, Adams, Gill, Harger, Wattles, and C. B. Robinson, bearing particularly upon the time to be allowed in Veterinary Colleges to graduates of Agricultural Schools, and the need of so many Veterinary Colleges throughout the country. Dr. Williams, in his remarks, referred to the statement made in Chicago at our last meeting, relative to the fact that many counties in Illinois did not have a single veterinarian. He wished to correct a false impression that might follow from this statement, by saying that there were many counties in the southern portion of Illinois where a veterinarian could not get a living if he had all the work there was to be done in three or four counties. The average value of horses in many of these counties would not exceed $10.00 per head. The question of Civil Service examinations held by the Government was also touched upon by several of those who discussed the papers.

Following this, the report of the Committee on Diseases was called for and read by the Chairman, Dr. Leonard Pearson. It was discussed by Drs. Harger, Clement, Pearson, Salmon, Faust, Tait Butler and Williams. The report pointed strongly to the need of greater original work in the line of a better understanding of such diseases as influenza, cerebro-spinal meningitis, osteo-porosis, Texas fever, azoturea, puerperal apoplexy and many others.

The report of the Publication Committee was then offered by Dr. A. T. Sellers, second member of the committee. Reference was made to the cost of the annual publication ; also future employment of stenographers, and a high compliment was paid to the fine production by the printers employed by the Association. The President, in drawing attention to the report of the committee, desired, under the circumstances, an expression of opinion as to whether the Association should continue to annually print their transactions each year. The last year, including the stenographer's bill, amounted to almost $1,100.00. A vote was taken and it was unanimously decided to continue the publication of the transactions annually.

It was suggested that in the future in sending out the programmes that the names of the various members of the committees should be published in conjunction therewith. The Secretary was ordered to make a note of the same.

The Secretary then presented his report for the year, with a summary of the work that was performed and an item of the expenses incurred therewith, after which, at 6 P. M., the Convention adjourned until 10 o'clock Wednesday morning.

The second day's session convened at 10 A. M., President Hoskins in the chair. On roll call by the Secretary, the following members were present : Drs. Ackerman, Allen, Bachman, Barron, Bretherton, Bridge, Tait Butler, Cary, Clement, Conrow, Curtice, Dixon, William Dougherty, Eves, Faust, Formad, Gill, Goenter, HagenBurger, Hanson, Harger, Hartman, Hoskins, Kooker, Liautard, Lintz, J. P. Lowe, Lusson, Lyman, Mackey, Martenet, E. Mayhew Michener, W. B. E. Miller, Osgood, Paige, Parker, Pearson, A. F. Peters, T. B. Rayner, Reynolds, Rhoads, J. L. Robertson, J. D. Robinson, Salmon, Schwarzkopf, James Stewart, Trumbower, T. J. Turner, Williams, Winchester and Zuill.

New member present--C. H. Doepel.

Others present—Drs. A. F. Schrieber, Philadelphia, Pa., Jno. R. Hart, Philadelphia, Pa., J. H. Wattles, Kansas City, Missouri, H. J. Detmers, Columbus, Ohio, J. T. McAnulty, Philadelphia, Pa., M. E. Conard, West Grove, Pa., George B. Rayner, Philadelphia Pa., J. C. Bartholomew, Berwyn, Pa., J. T. Ferley, Philadelphia, Pa., C. B. Robinson, Washington, D. C., R. W. Hewitt, Bridgeton, N. J., Cullom, Pa., S. G. Dixon, Philadelphia, Pa., Mr. J. G. Holland, Chicago, Ill., Mr. D. M. Keller.

A letter was read from City Veterinarian Hart, extending an invitation to all those present to visit the Municipal Veterinary Hospital of the city.

A letter was read from the Rev. William I. Nichols, of the Spring Garden Unitarian Society, referring to the fashion of docking horses' tails. The letter was referred to the Comitia Minora by the President.

The Secretary then reported that the Comitia Minora had favorably recommended Drs. M. H. McKillip, M.D., V.S., Chicago and Ontario V. C., 1639 Wabash Ave., Chicago, Ill. Vouchers, J. M. Wright and J. S. Butler; L. A. Merillat, V.S., Ontario V. C., 1639 Wabash Ave., Chicago, Ill. Voucher, J. M. Wright, for membership. Drs. Lemuel Pope, Jr., M.D.V., V.D., Harvard University, Portsmouth, N. H. Vouchers, C. P. Lyman and F. H Osgood; D. S. White, D.V.M., Ohio University, Columbus, Ohio. Voucher, Leonard Pearson; R. P. Lyman, M.D.V., V. D. Harvard University, 47 Mt Vernon St., Boston, Mass. Vouchers, C. P. Lyman and F. H. Osgood; H. J. Detmers, Veterinarian, Thierartzlichen Hochschule, Berlin, Columbus. Ohio. Voucher, Leonard Pearson, were also favorably recommended, and were unanimously elected to membership.

The Secretary then read the list of delinquents as follows: Drs. Carrick, Cherry and Prophett. On motion it was ordered that their names be dropped from the roll for non-payment of dues.

Charges were laid before the Comitia Minora, of violation of the code of ethics, by Dr. Winchester against Drs. Lee and Beckett of Boston, Massachusetts, in the nature of an illuminated calendar for general distribution. After some discussion, it was decided that Drs. Lee and Beckett be summoned before the Comitia Minora at the next meeting to answer to the same.

At the meeting in Chicago, Dr. Wilkinson's name had been reported, of Claremont, N. H., for violation of the code of ethics in advertising, and since then information had been afforded the officers that he also vended secret combinations of medicine, and assumed the title of D.V.S., which was not a degree granted to him by the college of which he claimed to be a graduate of. He was recommended for dismissal, which recommendation was approved by the Association.

The various State Secretaries' reports were then called for. Dr. Cary's, of Alabama, being a verbal one, stating that he knew of but five qualified veterinarians in the State. He hoped during the year that they would be able to form a State Association there.

Dr. Robinson, of the District of Columbia, had failed to bring his report with him, and reported the existence of two colleges and a large increase in the number of district veterinarians.

In the report of Illinois, Dr. Wilson stated that professional work had been so very quiet in Illinois that there seemed to be little in that direction to make any report of.

Dr. Archibald of California reported the enactment of a law regulating the practice of veterinary medicine and surgery in California. It created a State Board of Veterinary Examiners, and a great deal of good was looked forward to as the result of this legislation. The Veterinarians of California were also very active, looking toward a better Veterinary Sanitary Police for the entire State.

Dr. Greeson, of Indiana, afforded the Secretary a list of graduates from that State.

Dr. Harrison of Kansas gave a brief report of the infectious and contagious diseases in the State, reporting Glanders and Actinomycosis ; and also announced the formation of the Missouri Valley Veterinary Medical Association, including the veterinarians of Missouri, Kansas, Nebraska and Iowa. He also included a letter of State Veterinarian Pritchard of Kansas, which reported several outbreaks of Texas fever, about 30 cases of supposed Rabies, 57 cases of Glanders, 8 of Tuberculosis, 11 of Actinomycosis, 7 of Button Farcy and 50 of Keratitis in three outbreaks among cattle, and nine cases in an outbreak in a herd of horses. He looked upon it as contagious.

Dr. Martenet, Secretary for Maryland, reported the condition of the profession in his State, reporting the new graduates that had entered during the year, and also of the efficacy of the law which had recently been passed in Maryland, establishing a Veterinary Examination Board.

Dr. Parker, for Massachusetts, in his report gave a brief synopsis of the important action and the valuable points incorporated in a recent law passed in Massachusetts, looking toward the control and eradication of Tuberculosis and other contagious diseases among domestic animals, calling attention to its value in providing for a more thorough inspection of the dairies and sources of milk production.

In the absence of a report from Secretary Meyer, of Missouri, a report was made by Dr. Turner showing the general condition of the profession in Missouri.

A brief verbal report was made by Dr. Williams, of Montana, quoting the fact that it is one of the largest States in the Union, and at the same time has only five qualified veterinarians in the State, referring to the fact that but three kinds of livestock were contained in the State ; horses, cattle and sheep The State endeavors to destroy all cases of any dangerous contagious diseases, and allows no compensation to the owners. Veterinary Science is taught in Agricultural Colleges in Montana but only so far as to make the students better stock raisers, thereby gradually increasing the breeding of a better grade of stock.

Secretary Edwards, of Iowa, commented upon the deterioration in value of stock, and horses in particular stating the fact that many heavy, draft horses and others of slight value were actually being destroyed for their hides, and that in the breeding districts there was a very great reduction in the number of horses being bred. He estimated that there would be a difference of 25 per cent. in the number of horses in the State a year hence. Referring to the two Veterinary Schools in the State, the Iowa Veterinary College, at Des Moines, a two-year school, as being inadequately equipped for the proper instruction of students. Also referring to the high entrance examination required by the Veterinary Department of the Iowa State College. It being so situated as not to be dependent upon fees for its support and maintenance. He estimated that there were 135 graduates in the State of Iowa, and noted the general good standing of the men, and their high recognition in their methods of practice and advertising. He referred to the two Associations existing in Iowa and to the work they were doing.

Dr. H. J. McClellan, in presenting his report for Pennsylvania referred to the work being done in Pennsylvania by the five local Associations working in harmony with the State Association. Reporting the State Association with having 124 active members, though there were still thirty-six counties in the State not represented in the Association. There were over 300 members in the five Associations. He referred to the proposed legislation looking to the establishment of a Live-stock Sanitary Commission and a State Veterinary Board of Examiners, and hoped there would be a generous support of these measures by the entire profession, and particularly those of Pennsylvania. He criticised the present method of dealing with dangerous diseases among live-stock in the State, and lack of State recognition of the Veterinary profession.

A brief report of the Committee on Army Legislation was made by Dr. F. L. Killborne, announcing again their inability to successfully accomplish legislation in this direction. He again laid stress, as had been done in other reports, that until there was a complete unanimity of all those concerned as to what should be the form of legislation granted, that there was little prospects of success attending any legislative efforts.

The reports of the several special committees were then called for, and Vice-President Clement taking the Chair, Dr. Hoskins, as Chairman of the Committee on Congress of Colleges, reported briefly the successful accomplishment of their purpose, referring to the convening on July 14th. at Buffalo, and a more recent meeting in Philadelphia, on the 17th, with the following additional colleges represented: the Ohio State University, the Kansas City College, the American, National College, and the McGill University, announcing that an organization had been formed of the Faculty of the various Schools of North America, and the adoption of the recommendations made at Buffalo, which have already been printed in these columns. He called upon the permanent President, Dr. Lyman, to further supplement the report of the committee.

Dr. Lyman spoke of the great encouragement that the movement had met with, and already felt that a great deal had been gained by this commingling of these various representatives, and a better understanding had been accomplished among the Schools.

In the discussion of the report, Dr. Harger highly complimented the work of the committee, and also the formation of this Association of Veterinary Faculties.

Dr. Osgood, referring to the work of this Convention, stated that while there were ten schools represented there had been no resolution passed except by the unanimous approval of all present, and that the constitution of the new organization had been signed by the representatives of these ten schools.

On receiving the report, it carried with it the acknowledgment of the great obligations of the Association for the work done by their committee.

The next special committee was the Committee on Act of Incorporation. Dr. Lyman, as Chairman, made his report, including a number of letters from various members of the United States Senate, in which body the bill had been presented. He stated the bill had been referred to the Committee on Military Affairs, and later had been referred to the Committee on Agriculture and Forestry. Owing to the tariff bill being paramount to all other questions, they were unable to secure favorable consideration at this session of Congress, and recommended that the committee be continued and the matter pressed at the next session.

A report of the Committee on Revision of Constitution and By-Laws was then called for, and Dr. Miller, Chairman, responding, read the various suggested changes, and they were taken up. Among the changes was the establishment of a better method for the payment of all bills and expenses of the Association. The initiation fee was, after considerable discussion, allowed to remain at $5.00, and the annual dues were raised to $5.00; also that all initiation fees should everafter accompany the application for membership, otherwise said applications not to be considered. The annual meetings of the Association were changed to the first Tuesday and the two following days in September of each year. This was done because it would be more convenient to all those connected with Agricultural Colleges, and equally as conducive in attendance to those connected with the teaching staff of all schools. Another change of significance was the establishing of three Vie-Presidents, one assigned to each of the three sections of time limit; also the Secretary was made, by virtue of his office, a member of the Publication Committee. A number of minor changes were made, all looking toward a better administration.

Telegrams of regret at enforced absence were read from Drs. Hinkley and Wm. H. Lowe.

After which the meeting adjourned at 1.30 to reconvene at 2 P. M.

The Convention reconvened at 2 P. M., and Dr. Harger was accorded the privilege of the floor to explain a photograph of a curious case that had been before his department at the Veterinary Department of the University of Pennsylvania. It was of a calf with the heart located in the anterior cervical region of the neck, separated only from the skin by some adipose tissue.

Dr. Osgood, as Chairman, then reported the following resolutions on the death of Dr. John S. Saunders:

*Whereas*, It becomes the painful duty of the Committee to announce to the Association the death of an old time member, John S. Saunders, be it

*Resolved*, That in his removal we are deprived of further association with one who, respected by all, was particularly dear to the older members, who well remember his hearty greetings and thoroughly appreciated his many good qualities.

He was a hard and faithful worker and ever ready to extend a helping hand. He was honest, sincere, and strove always to elevate his profession. His genial nature endeared him to all, and we mourn the loss of a beloved associate; be it

*Resolved*, That a copy of these resolutions be published in the veterinary journals, a copy forwarded to the family of the deceased, and a copy spread upon the records of this Association.

F. H. Osgood, Chairman.
J. L. Robertson,
L. H. Howard.

And by Dr. Clement, as Chairman, in relation to the death of Dr. Michener:

*Whereas*, Death has removed from among us one of our oldest members, Dr. Chas. B. Michener, and

*Whereas*, We feel deeply the loss sustained by us in the early demand of one with whom we have so long been associated; be it

*Resolved*, That we hereby express our deep felt sorrow in the loss of such a genial companion, untiring worker and devoted friend; be it

*Resolved*, That a copy of these resolutions be spread upon the records of our Association, and forwarded to the family of the deceased and to the veterinary journals for publication.

A. W. Clement, Chairman.
Jno. Faust,
W. L. Zuill.

The committee on a better Nomenclature of Swine Disease and Hog Cholera made their report in which they seemed to be of the unanimous opinion that it would not be well to make any changes at this time until the pathology of the diseases was more definitely decided.

The report was discussed by Drs. Salmon and Clement.

The nomination and election of officers then followed and the following was the result :—

Dr. Harger nominated W. Horace Hoskins for President. Seconded by Dr. Winchester, and Secretary casting the vote, Dr. Hoskins was unanimously elected President.

Nominations for Vice-President were now open.

Drs. Clement and Pearson were nominated, but withdrew.

Drs. Turner for the central, Williams for the western, and Winchester for the eastern districts were nominated and elected.

Drs. Leonard Pearson, L. H. Howard and J. W. Adams were nominated for Secretary. Dr. Howard withdrew. A vote was then taken and Dr. Pearson was declared elected.

Dr. Jas. L. Robertson was nominated for Treasurer and elected.

Dr. Cooper Curtice, at this point, offered a suggestion that a Secretary be yearly appointed to report a synopsis of the work done in Veterinary Science and in the various Agriculture schools and the Bureau of Animal Industry. It was referred to the Comitia Minora.

A paper by Dr. Harger was then called for entitled "Neurectomy," which was a very valuable contribution. This was very favorably commented upon by Dr. Salmon, who in his remarks thought that it was in such lines of work that the Association might yearly increase its usefulness and value to the profession at large. Drs. Conrow, Ackerman, Parker, Winchester, Clement, HagenBurger, Eves, Reynolds, and Tait Butler discussed the paper from many points.

The President then announced that they would take up the unfinished discussion of the paper of Dr. Harrison, read at Chicago last year, on "A New Method of Treating Periodic Ophthalmia by Surgical Interference," and asked Dr. Harger to open the discussion. The paper was further discussed by Drs. Cary, Winchester, Clement, Curtice, Parker, Tait Butler, Reynolds and Turner, bringing out many points in connection with the disease. A general lack of approval seemed to prevail as to the wisdom of performing a paracentesis for this disease.

After which the meeting adjourned at 6 P. M.

The third day's session convened at 10 A. M., with President Hoskins in the chair. On roll call the following members responded to their names :—Drs. Ackerman, Allen, Bachman, Brenton, Bridge, Budd, Tait Butler, Cary, Clement, Conrow, Curtice, Dixon, Eves, Faust, Formad, Gill, Goentner, HagenBurger, Hanson, Harger, Hartman, Hickman, Hoskins, Howard, Liautard, Lintz, Lusson, H. J. McClellan, Mackey, Martenet, W. B. E. Miller, Ogden, Paige, Parker, Pearson, A. F. Peters, J. B. Rayner, T. B. Rayner, Reynolds, Rhoads, J. L. Robertson, Salmon, Schwarzkopf, T. Smith, Jas. A. Stuart, Trumbower, T. J. Turner, Williams and Zuill.

New members present :—Drs. Doepel, George, Hewitt and Weicksel.

Others present :—Drs. S. G. Dixon, Phila., Pa.; M. E. Conard, West Grove, Pa.; M. W. Drake, Phila., Pa.; J. C. Bartholomew, Berwyn, Pa.; Walter L. Hart, Phila., Pa.; Jno. R. Hart, Phila., Pa.; H. B. Felton, Olney, Pa.; J. T. Ferley, Phila., Pa.; J. C. Foelker, Allentown, Pa.; J. T. McAnulty, Phila., Pa.; C. E. Fouse, Phila., Pa., and W. G. Benner, Doylestown, Pa.

The Secretary read the minutes of the meeting of the Comitia Minora. On motion they were adopted.

The following applicants for membership were favorably recommended and on motion elected :—Drs. A. G. Wicks, V.S., Ont. V. C., 429 Liberty St., Schenectady, N. Y.; Vouchers, N. P. Hinkley, B. P. Wende, Jno. Faust. Jno. W. Adams, V.M.D., Univ. Pa., 3423 Walnut St., Phila.; Voucher, W. Horace Hoskins. A. H. French, M.D.C., Chicago V. C., Aberdeen, Miss.; Voucher, C. A. Cary. Chas. E. Clayton, D.V.S., A. V. C., Bridgeport, Conn.; Vouchers, W. J. Coates, T. J. Turner. A. MacDonald, V.S., Ont. V. C., Fort Du Chesne, Utah; Voucher, Jas. A. Waugh. F. A. Donaldson, V.S., Ont. V. C., 305 N. Mansal St., Richmond, Va.; Vouchers, Geo. C. Faville, E. P. Niles. J. H. Wattles, D.V.S., Chicago V. C., Kansas City, Mo; Voucher, S. Stewart. S. E. Lloyd, D.V.S., A. V. C., Baltimore, Md.; Vouchers, Wm. Dougherty, A. W. Clement. A. J. Burkholder, D.V.S., A. V. C., Staunton, Va.; Vouchers, A. Liautard, W. J. Coates. S. S. Brooks, V.S., N. Y. C. V. S., Hamilton Ave. and 16th St., Brooklyn, N. Y.; Voucher, E. B. Ackerman. Wm. C. Siegmond, D.V.S., A. V. C., 1918 Wilkens Ave., Baltimore, M. D.; Vouchers, W. H. Martenet, J. L. Robertson.

The Comitia Minora reported the following resolution in regard to the question of docking horses' tails, which, after some discussion, were adopted :—

*Whereas*, We, the United States Veterinary Medical Association, do condemn the operation of "docking" horses' tails as an operation of fashion, believing that nature intended them as a defense against insects, etc.,

But so long as it is demanded as an operation of fashion, we believe that it should be performed by skilled Veterinarians, who are sure to perform it with greater care, less pain and suffering to the animals, and we cannot unqualifiedly condemn those who perform the operation under such conditions.

The Finance Committee, who had been working upon the financ'al condition of the Association, made the following report as to the finances of the Association :

JAS. L. ROBERTSON, TREASURER, IN ACCOUNT WITH THE U. S. V. M. A.

| | | |
|---|---|---|
| Amount in the Treasury, Oct., 1893.......... | | $875 73 |
| Less Error in Account, 1893..... ........... | $ 9 25 | |
| Less Amount Remaining in Secretary's Hands, 1893............. ................. | 108 33 | 117 58 |
| Total Amount in Treasury, October, 1893.. | | $757 15 |
| Dec. 14, 1893, To Wm. J. Dornan, Printer............ | $497 72 | |
| May 7th, 1894, To A. H. Baker, for Stenographer....... | 250 00 | 747 72 |
| | | $ 9 43 |
| To Amount Received from Secretary, T. J. Turner........... ................ | | 175 65 |
| Amount Received from Dues....... ....... | | 15 00 |
| | | $200 08 |

| | | |
|---|---|---|
| Sept. 13th, 1894, | | |
| To Wm. J. Dornan, Printer............ | $200 00 | 200 00 |
| Balance in Bank........................ | | 08 |
| Balance in Secretary Hoskins' Hands. ....... | | 108 33 |
| As Receipts from Chicago Meeting, October, 1893................ ............. | $332 30 | |
| To Expenses of President, Per Statement..... | 203 77 | |
| Balance Due Treasurer................. .... | | 128 53 |
| Total Resources......................... | | $236 94 |
| To Total Receipts, Secretary Turner, to Sept. 14th, 1894....,...................... | $575 50 | |
| To Total Expenses, Secretary Turner, to Sept. 14th, 1894........... ........... | 189 40 | |
| | $386 10 | |
| Less Amount Paid Treasurer ............... | 175 65 | |
| | $210 45 | |
| To Balance in Hands Secretary Turner........ | | 210 45 |
| Total Resources................ ....... | | $447 39 |

The members who had been requested by the Comitia Minora to remove the violations of the code of ethics, in regard to objectionable forms of advertising, that were written to by the secretary, with one dissenting, had indicated their willingness to rectify the same at once.

The Comitia Minora recommended the acceptance of Dr. Gadsden's resignation, per his request, which was approved by the association.

Dr. I. N. Krowl was recommended for suspension for non-payment of initiation fee and dues, and said recommendation was approved by the association.

Dr. W. T. Russell, who had refused to correct the objectionable form of advertising at variance with the code of ethics, was recommended for expulsion. Said action was approved by the association.

Dr. E. B. Ackerman, for the State of New York, was then asked to read his report, in which he referred to the colleges of his State, and spoke of the prospectve school at Cornell, which was to be under the direction of Prof. Law. Referring to their State Society, with its more than one hundred members, and of the active work they were doing in establishing local societies in various counties in the State ; also referred to the new County Society of New York, and of the active work being done by that organization. He referred to the establishment of a Tuberculosis Commission in New York, and of the number of animals that had been examined and destroyed ; also referred to the reports of the veterinarians of New York and Brooklyn State Boards of Health, and of the fact that 300 cases of glanders and farcy had come under their notice during the past year.

The other State reports, such of them as contained interesting matter, were ordered to be made a part of the proceedings of the association.

The paper of Dr. W. L. Williams was then called for and read, on "The Influence of Climate and Other Environments on the Distribution and Character of Disease." It proved one of the most valuable papers the association has ever had the privilege of listening to, and rather than comment upon it here, we think that not a single member of the profession in the country should fail to read it, and particularly the members of the association, as an incentive for the yearly preparation of such papers, which add so much to the pleasure and knowledge of the profession, on conditions of such great importance.

The paper was discussed by Drs. Trumbower, Schwarzkopf, Harger, Curtice, Salmon, Peters and Rhoads, bringing out more clearly many valuable points of the paper.

The topic of "Tuberculosis" was then taken up by a paper by Dr. Faust, which after reading of the same, adjournment took place for lunch.

The meeting reconvened at 2 P. M., and the Comitia Minora favorably reported the following applicants for membership:

Drs. R. W. Hewitt, D.V.S., A. V. C., 15 Warren Street, Bridgeton, N. J.; voucher, H. D. Hanson. J. O. George, A.B., A.M., D.V.S., A. V. C., Camden, N. J.; voucher, H. D. Hanson. B. F. Bachman, V.M.D., Univ. of Pa., V.D., Stroudsburg, Pa.; voucher, Leonard Pearson. Paul Fischer, D.V.M., Ohio Univ., Columbus, Ohio ; voucher, Leonard Pearson.

On motion they were unanimously elected to membership.

Dr. Pearson then presented his views on the question of tuberculosis, calling attention to many important points in regard to dealing with this disease.

He was followed by Dr. Trumbower, as to "How We Can Control the Prevalence of Tuberculosis Among Cattle."

The President then announced the subject open for discussion, which was participated in by Drs. Parker, Clement, Trumbower, Salmon, Bridge, Allen, Peters, HagenBurger and Curtice, most of whom contributed some additional information commending the value of tuberculin as a diagnostic agent.

Two proposals for additional changes in the by-laws were referred to the Comitia Minora.

A vote of thanks was then extended to the Trustees of the Academy of Natural Science for the courtesy in the use of their rooms for the sessions of the Convention.

A vote of thanks was also extended to the Press of Philadelphia for their excellent reports of the daily proceedings of the Convention.

A vote of thanks was extended by the visiting members to the Philadelphia members for their kind reception and entertainment.

The President directed that all other papers which were offered should be published in conjunction with the transactions of the association, after which the Convention adjourned, and, in a body, were taken by coach to the Veterinary Department of the University of Pennsylvania, Philadelphia Veterinary Sanitarium and Zoölogical Garden, returning over a portion of the city.

At 8 P. M. about forty met at the banquet table at the Colonnade Hotel, and fittingly closed the Convention by one of the most pleasant and agreeable meetings of this kind ever held by the association. The responses to the various toasts were heartily enjoyed by all those who sat at the table.

(Reported by W. H. H.)

