# Correction

**Published:** 1895-01

**Authors:** 


Correction.—We regret very much that through an incomplete report of the stenographer the following names of gentlemen elected to membership at the last annual meeting of the United States Veterinary Medical Association were not noted in the report in the November issue of the Journal: Jno. H. Adamson, M.D.C., Chicago V. C., 1327 H Street, N. W., Washington, D. C.; Don C. Ayer, D.V.S., A. V. C., Omaha, Nebraska; W. H. Dalrymple, M.R.C.V.S. Glasgow, Atlanta, Ga.; M. J. Treacy, M.R.C.V.S. Edinburgh, Fort Meade, S. D.

